# Sensitivity Enhancement of Hybrid Two-Dimensional Nanomaterials-Based Surface Plasmon Resonance Biosensor

**DOI:** 10.3390/bios12100810

**Published:** 2022-09-30

**Authors:** Nurzad Zakirov, Shaodi Zhu, Aurélien Bruyant, Gilles Lérondel, Renaud Bachelot, Shuwen Zeng

**Affiliations:** 1 Light, Nanomaterials & Nanotechnologies (L2n), CNRS-ERL 7004, Université de Technologie de Troyes, 10000 Troyes, France; 2 Department of Biomedical Engineering, The Chinese University of Hong Kong, Shatin, Hong Kong

**Keywords:** biosensors, graphene, surface plasmon resonance, plasmonics

## Abstract

In this work, we designed structures based on copper nanosubstrate with graphene and two-dimensional transition metal dichalcogenides (TMDC) in order to achieve an ultrasensitive surface plasmon resonance biosensor. This system contains seven components: SF11 triangular prism, BK-7 glass, Chromium (Cr) adhesion layer, thin copper film, layers of one of the types of transition metal dichalcogenides: MoS_2_, MoSe_2_, WS_2_ or WSe_2_ (defined as MX_2_), graphene, sensing layer with biomolecular analyte. Copper was chosen as a plasmonic material because it has a higher conductivity than gold which is commonly used in plasmonic sensors. Moreover, copper is a cheap and widespread material that is easy to produce on a large scale. We have carried out both theoretical and numerical sensitivity calculations of these kinds of structures using the Goos–Hänchen (GH) shift method. GH shift is lateral position displacement of the p-polarized reflected beam from a boundary of two media having different indices of refraction under total internal reflection condition and its value can be retrieved from the phase change of the beam. The SPR signal based on the GH shift is much more sensitive compared to other methods, including angular and wavelength scanning, due to much more abrupt phase change of the SPR reflected light than its intensity ones. By optimizing the parameters of the SPR sensing substrate, such as thickness of copper, number of layers of 2D materials and excitation wavelength, we theoretically showed an enhanced sensitivity with a detection limit 10^−9^ refractive index unit (RIU).

## 1. Introduction

The surface plasmon resonance (SPR) technique is considered as the most successful plasmonic application so far [1]. Among all biosensors, surface plasmon resonance (SPR) sensor exhibited several key advantages that cannot be achieved by other sensing techniques such as real-time detection, high signal-to-noise ratio and wide dynamic range with ultra-high sensitivity for small chemical and biological markers [2,3]. The path of the laser beam used in the technique does not pass the sample, making it available for the detection in complex samples like blood or plasma. Detection time is also better: around 10 min compared to 72 h for polymerase chain reaction (PCR) tests, and it has less preparation time. Another cost-effective advantage of SPR is the possibility to use sensor chips more than once. Sensor chips directly influence the quality of the result and serve as an essential component of biosensing.

The major barrier to broad application of plasmonic devices is the absence of available and inexpensive materials which can be easily manufactured. Most of the current SPR biosensors are based on gold thin film. However, the sensitivity for the continuous gold with thickness around 45–50 nm is not sufficient for low concentration (1 fM to 1 nM) of biomarkers detection. Recently, many research groups have investigated novel nanomaterials capable of flexible tuning the optical properties of the plasmon resonance at the sensing interface [4,5].

The stability of plasmonic materials was reached by investigating new methods of protection from oxidation. Copper (Cu) exhibits better electrical conductivity compared to gold and SPR systems can be made by using copper substrate [6]. This can be explained as follows: when atoms get larger, with a high number of electrons the influence of the positively charged core of the atom on the furthest electrons is weak. However, for atoms ending with d-orbitals, including copper which has only 29 electrons, the electrical conductivity is higher than some metals containing more electrons, such as gold with 79 outer electrons.

Graphene (~0.34 nm), which is a single carbon atomic layer that can exist at room temperature, was firstly obtained in 2004. The semi-metal properties of graphene allow the tuning of the sensitivity properties of sensing devices. When graphene layers are deposited on a metal substrate, the electron charge transfer between the graphene and gold substrate results in a large near field amplification, thus a strong plasmonic coupling will happen at the sensing areas. [7]. Work done by Geim and Novoselov made it possible to produce graphene in comparingly large scale for practical use and was awarded the Noble prize in physics in 2010 [8]. A large area of graphene layers ranging from 1 cm to 90 cm can be grown with chemical vapor deposition (CVD) method [9]. According to CVD method, graphene is first deposited on the copper substrate. In addition, copper (Cu) exhibits a higher conductivity than gold and can be used as plasmonic material. Graphene layers can also protect the Cu surface from oxidation. Cu was covered by graphene and proved by the experiment that it can retain plasmonic properties even after half of a year [10].

We have designed a hybrid SPR sensing nanostructures based on graphene and other types of 2D materials—transition metal dichalcogenides (TMDCs). TMDCs can enhance the performance of biosensors by precisely tuning the optical absorption of the sensing substrate. 2D materials used here are intended to improve the sensitivity of the system, as well as protect the metal surface from deterioration. For graphene layers, it can enhance the attachment of molecules due to the pi stacking forces. Maurya, J. B., and Prajapati Y. K. first described the reflectivity of Cu film with monolayers of MoS_2_ and graphene [11]. They provided the angular sensitivity of this configuration, i.e., SPR angle shift corresponding to refractive index change of the sensing media. In addition to Cu film, the reflectivity and angular sensitivity of Au, Ag and Al films with MoS_2_ and graphene monolayers were discussed in this work as well. Rather than interrogating the change of SPR angle, our work mostly focuses on the phase and phase-singularity enhanced GH shift. Moreover, we explored different MX_2_ materials more than only MoS_2_, such as MoSe_2_, WS_2_ and WSe_2_ in combination with graphene. Here, we have proposed a graphene-TMDC hybrid nanostructures for the SPR sensitivity improvement. Theoretic work was done using TMDC materials in SPR biosensors with Au-Ag bimetallic film [12] and it showed that graphene with 2 layers WS_2_ can improve the optical absorption, resulting lower dip in the reflectance curve under resonance condition. Additionally, the improved sensors are of great importance in the emerging field of edge sensing and computing, as described by Passian A. and Imam N. [13].

The SPR reflection spectra of this configuration were theoretically modelled by using the Transfer Matrix Method (TMM) and Fresnel equations. These equations and TMM are known to be used to study multi-layered SPR structures under excitation condition [14]. In this project, another key novelty is based on the SPR phase singularity-enhanced lateral position shift (Goos-Hänchen (GH) shift) [15]. Under the resonance condition, the large position shift due to the strong plasmon resonance coupling is only generated by the p-polarized light corresponding to the TM waves, where s polarization is taken as a reference signal. It will also significantly improve the sensing configuration’s signal-to-noise ratio. Surface plasmon resonance phenomenon can produce a GH shift larger than 50 times of the incident wavelength [16]. Moreover, it is possible to measure this position shift by a quadrant position sensor. Thus, if one can achieve an ultra-low reflection in the SPR dip, a steep optical phase jump can be obtained [17]. In general, the phase in electromagnetic mechanisms is determined by oscillation cycle of the signal. Optical phase signal can be extremely large and sharp when the light intensity is reaching zero, which means the lower the intensity at the sensing interface, the higher the phase signal change. Thus, by tuning the plasmonic nanostructures, we can achieve the darkness and enhance the phase singularity [18]. This steep phase signal change will result in a large position shift in the x-direction. An ultra-high sensitivity by 2–3 orders of magnitudes can be achieved compared to current SPR sensors based on angular or wavelength scanning [15,17]. Because of advantages of GH shift in optical sensing and precision measurement, it has been widely discussed in the chemistry and sensors area [19,20].

Here we have reported new SPR sensing substrate based on copper enhanced by TMDC materials and graphene layers. In this system, 2D materials are used to improve light absorption giving sufficient energy to electrons. Graphene is also used for enhancing the adsorption of target biomolecules through the force of pi-stacking.

The highest sensitivity value observed equals to 2.26 × 10^6^ µm/RIU and it was obtained with TMDC material—WSe_2_ in combination with 2 layers of graphene under the excitation wavelength of 1024 nm in the near infrared region. The 1024 nm excitation wavelength results the highest sensitivity value for WSe_2_. The use of higher excitation wavelengths has an appreciable effect on the width of the resonance curve. This is one of the reasons why (near-) infrared SPR experiments are attracting attention. The width of the resonance curve is mainly determined by the complex value of the metal’s dielectric constant [21]. For the copper substrate, the 1024 nm excitation wavelength results a large imaginary part. Moreover, for graphene, the imaginary part of its refractive index gets larger with increasing the wavelength.

## 2. Methods

**Figure 1:**
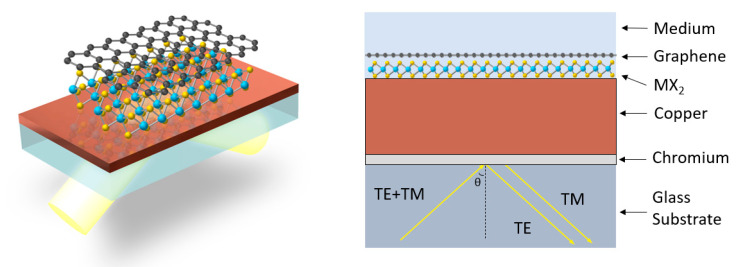
Schematic picture of Cu—TMDCs—graphene enhanced SPR biosensor system. The difference of GH shift for TM and TE waves are measured in order to improve the signal to noise ratio since the signals for TE waves can be used as a reference.

The proposed sensing configuration is consisted of the following components: SF11 triangular prism, BK-7 substrate, a chromium layer, a thin copper film, layers of one of the types of transition metal dichalcogenides: MoS_2_/MoSe_2_/WS_2_/WSe_2_ (defined as MX_2_), a graphene layer for capturing the targeted sample molecule, and a sensing layer with biomolecular analyte. Kretschmann’s attenuated total reflection (ATR) structure is chosen for this surface plasmon resonance sensor model. An illustration of the configuration is in Figure 1 [22].

In surface plasmon detection systems there are three main methods of detections:

- Angle interrogation;
- Wavelength interrogation;
- Phase change measurements;

The GH shift is in a higher order function of the optical phase, as shown in Equation (8). The lateral position shift was theoretically retrieved from the phase modulation. The reflectivity of the configuration was found from the Fresnel equations, describing the multi-layered plasmonic structures under excitation condition. Chromium adhesion layer’s thickness was chosen as 1.5 nm. The results were obtained using several incident wavelengths both in visible and near-infrared regions: 600 nm, 633 nm, 660 nm, 785 nm, and 1024 nm. Structures with varying numbers of TMDC layers were covered with 0, 1 and 2 layers of graphene.

### 2.1. Reflectivity (R_p_) and Phase (ϕ_p_) Calculations

Recently, phase shift detection method was implemented to significantly improve the sensitivity of SPR biosensors. Many experimental schemes for phase shift detection from the reflected light have been demonstrated [23].

To study the phase (ϕ_p_) and reflectivity (R_p_), the transfer matrix method (TMM) and the Fresnel equation for multilayer systems are taken. The system consists of coatings situated parallel to each other. Transmission matrix’s structure is defined as M in equations. Calculations for the light with p-polarization are made as follows [24]:

(1) $$M=\prod_{k=2}^{N-1}M_{k}=\left[\begin{array}{cc} M_{11} & M_{12}\\ M_{21} & M_{22} \end{array}\right]$$

with

(2) $$M=\left[\begin{array}{cc} \cos\beta_{k} & \left(-i\sin\beta_{k}\right)/q_{k}\\ -{\mathrm{iq}}_{k}\sin\beta_{k} & \cos\beta_{k} \end{array}\right]$$

where

(3) $$q_{k}={\left(\frac{\mu_{k}}{\varepsilon_{k}}\right)}^{1/2}\cos\theta_{k}=\frac{{\left(\varepsilon_{k}-n_{1}^{2}\sin^{2}\theta_{1}\right)}^{1/2}}{\varepsilon_{k}}$$

and

(4) $$\beta_{k}=\frac{2\pi }{\lambda }n_{k}\cos\theta_{k}\left(z_{k}-z_{k-1}\right)=\frac{2{\pi d}_{k}}{\lambda }{\left(\varepsilon_{k}-n_{1}^{2}\sin^{2}\theta_{1}\right)}^{1/2}$$

Here, n_k_ corresponds to refractive index of k layer (Appendix A). ε_k_ corresponds to the dielectric constant. d_k_ is the thickness of a particular layer. ϴ_1_ is the incident angle of the light and λ is the excitation wavelength. The reflection coefficient is indicated as r_p_, index “p” means belonging to the p-polarization and has a relation:

(5) $$r_{p}=\frac{\left(M_{11}+M_{12}q_{N}\right)q_{1}-\left(M_{21}+M_{22}q_{N}\right)}{\left(M_{11}+M_{12}q_{N}\right)q_{1}+\left(M_{21}+M_{22}q_{N}\right)}$$

where q_1_ is the value for the layer number 1, q_N_ is—the value for the last layer in Equation (3). Finally, the phase (ϕ_p_) and reflectance (R_p_) for the p-polarized light calculated:

(6) $$\phi_{p}=\arg(r_{p})$$

and

(7) $$R_{p}={\left|r_{p}\right|}^{2}$$

The graph of reflectance (R_p_) depending on the incident angle is so called SPR curve. The angle of the dip in SPR curve is known as the resonant angle of SPR (ϴ_SPR_). This angle changes with variation of refractive index related to a surface medium that is induced from binding of target molecules.

### 2.2. Goos-Hanchen (GH) Shift Calculations

The phase signal (ϕ_p_) undergoes a much sharper change compared to the change in angle of reflection or change of wavelength. GH shift (L_shift_) can be determined as a higher order phase variation. Because GH shift can be calculated from the phase change as shown in Equation (8). GH shift value is directly proportional to the phase derivative of the angle of incidence. It corresponds to the displacement of reflected light from the metal surface.

Quantitative measurement of the GH shift is challenging for total internal reflection (also called weak measurement), because of its low magnitude. However, when the incident angle at the surface plasmon resonance angle, this shift could be improved by 2–3 orders.

Upon excitation of surface plasmon resonance, the reflected light intensity for p-polarization of light is close to zero, and a phase jump was detected at the resonant angle. The phase jump is a sharp, quasi-vertical phase change corresponding to different angles of incidence under the SPR excitation condition. Phase delay at this exact point results in a large GH shift. Phase (ϕ_p_) and GH shift (L_shift_) are extremely sensitive to variations of ∆n_target_ from analyte in the media. In general, GH shift value is derived from the phase and angle of incidence ϴ_inc_ [25]:

(8) $$L_{\mathrm{shift}}=-\;\frac{\lambda_{\mathrm{inc}}}{2\pi \sqrt{\varepsilon_{1}}}\cdot \frac{\Delta \phi_{p}}{\Delta \theta_{\mathrm{inc}}}$$

The sensitivity based on GH shift method is defined as follows [15]:

(9) $$S_{\mathrm{GH}}=\frac{\Delta L_{\mathrm{shift}}}{\Delta n_{\mathrm{target}}}$$

According to Equation (9), the unit of sensitivity will be µm/RIU. The limit of detection of GH shift is better than values of refractive index measurements by other methods. RIU/nm is another unit of measure for the sensitivity.

GH shift values for 2 cases were calculated: with normal refractive index n, usually water, then with adding RI difference: n + ∆n. The value of ∆n was taken as 5 × 10^−5^ RIU. Then divide by ∆n the difference between these two GH shift values.

## 3. Results

### 3.1. Reflectance, Phase Change and GH Shift of WSe_2_ Structure

The sensitivity has been optimized considering three substrate parameters: the thickness of Cu, 2D materials type, and number of 2D layers.

**Figure 2:**
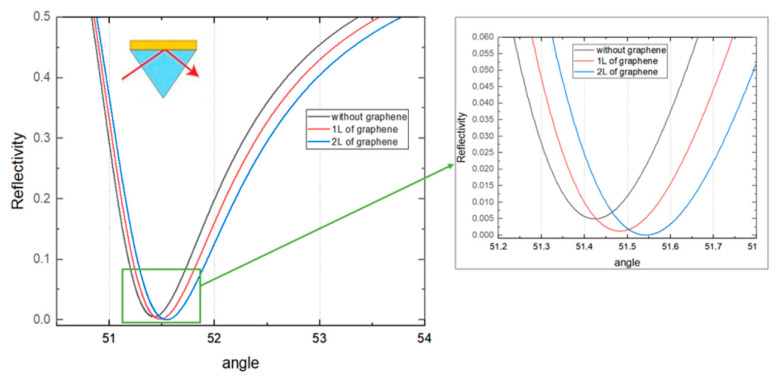
Reflectivity of the structure with 5 layers of WSe_2_ and 35 nm of Cu depending on the angle of incidence (degrees).

Different excitation wavelengths were studied to obtain the optimal performance. Among the five visible and near-infrared wavelengths, it was found that 1024 nm excitation wavelength shows the highest value which equals to 2.26 × 10^6^ µm/RIU or 4.43 × 10^−10^ RIU/nm. This structure consists of 35 nm Cu, 5 layers of WSe_2_ and 2 layers of graphene. In Figure 2, the reflectance curves are shown.

**Figure 3:**
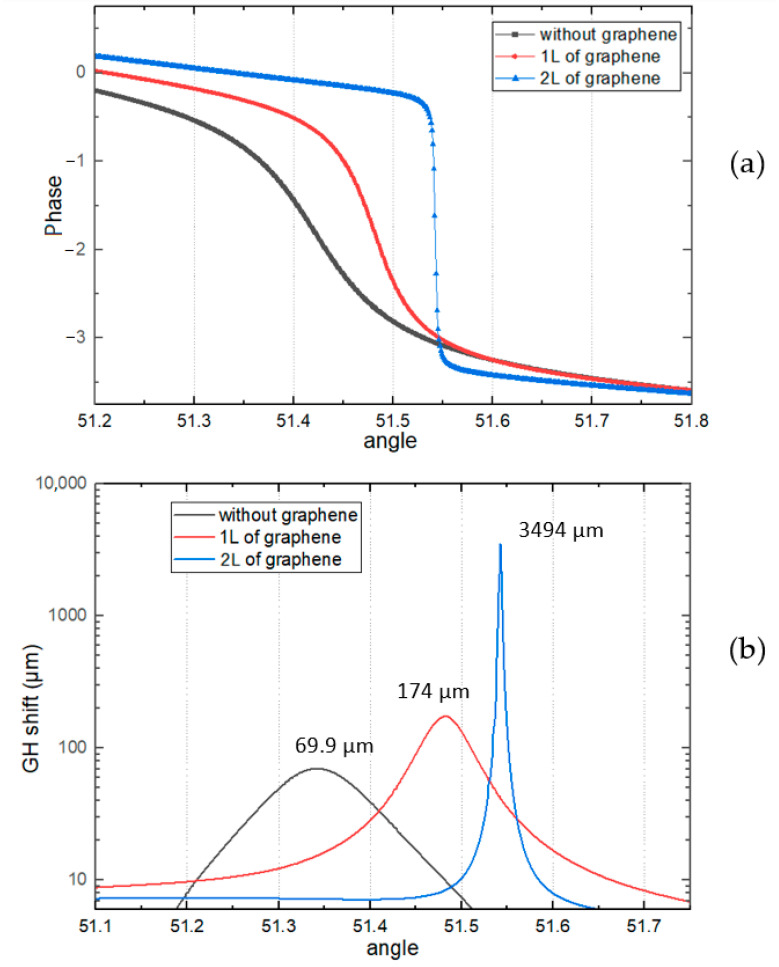
Phase calculations (**a**) and GH shift values (**b**) of the structure with 5 layers of WSe_2_ and 35 nm of Cu depending on the angle of incidence (degrees).

In Figure 3a the phase change is shown corresponding to the structures with different number of graphene layers. The quasi-vertical phase change in SPR angle is obtained for 2 layers of graphene, therefore it has a higher value for GH shift, as shown in Figure 3b.

The dip of the curve was shifted to the larger angle of incidence by increasing the number of graphene layers and having a lower value. The curve with the lowest reflectivity corresponds to the substrate with 2 layers of graphene. This dip in reflectance indicates the resonance angle for a given configuration and excitation wavelength. Most of the p-polarized light intensity was absorbed and thus leading to a strong resonance at the SPR angle when resonance occurred. Due to strong surface plasmon polariton coupling, minimum intensity reflected light was then detected.

### 3.2. Sensitivity with Graphene Layers

Firstly, structure with a copper covered by graphene was calculated. Number of layers of graphene was chosen as 2, because 1 layer will not be sufficient to protect the surface of copper from degradation, as some defects appear during the deposition process such a comparingly big surface.

As shown in the Table 1, the values for sensitivity vary from thickness of copper and wavelengths. For 30 and 50 nm of copper, the high values of sensitivity obtained mostly from the structure without graphene; for 35, 40 and 45 nm highest values are from 2 layers of graphene. Therefore, 35, 40 and 45 nm of thin copper film thicknesses were selected to achieve better performances. These calculations demonstrated the sensitivity enhancement by graphene. Moreover, copper surface oxidizes without a protective coating, resulting in much lower performance.

### 3.3. Sensitivity with TMDC Enhanced Model

**Figure 4:**
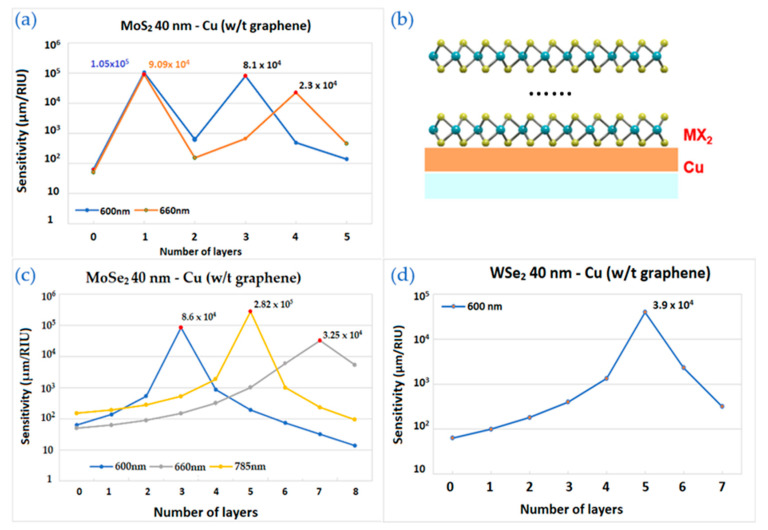
The sensitivity values of the enhanced model by number of TMDC layers with 40 nm of copper: (**a**) The wavelengths are 600 nm and 660 nm for MoS_2_ layers; (**b**) the schematic structure; (**c**) The wavelengths are 600 nm, 660 nm, and 785 nm for MoSe_2_ layers; (**d**) The wavelength is 600 nm for WSe_2_ layers and 40 nm of Cu.

**Figure 5:**
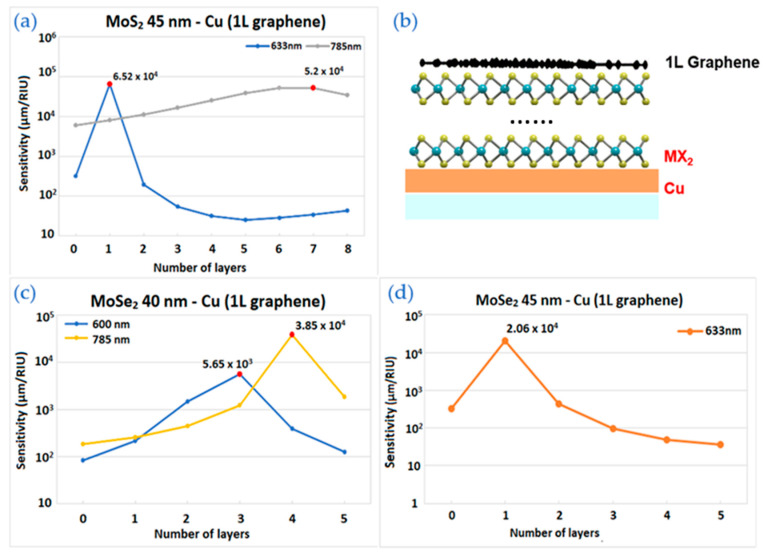
The sensitivity values of the enhanced model with 1 layer of graphene by TMDC layers and thickness of copper: (**a**) The wavelengths are 633 nm and 785 nm for MoS_2_ layers with 45 nm of copper; (**b**) the schematic structure; (**c**) The wavelengths are 600 nm and 785 nm for MoSe_2_ layers with 40 nm of copper; (**d**) The wavelength is 633 nm for MoSe_2_ layers with 45 nm of copper.

**Figure 6:**
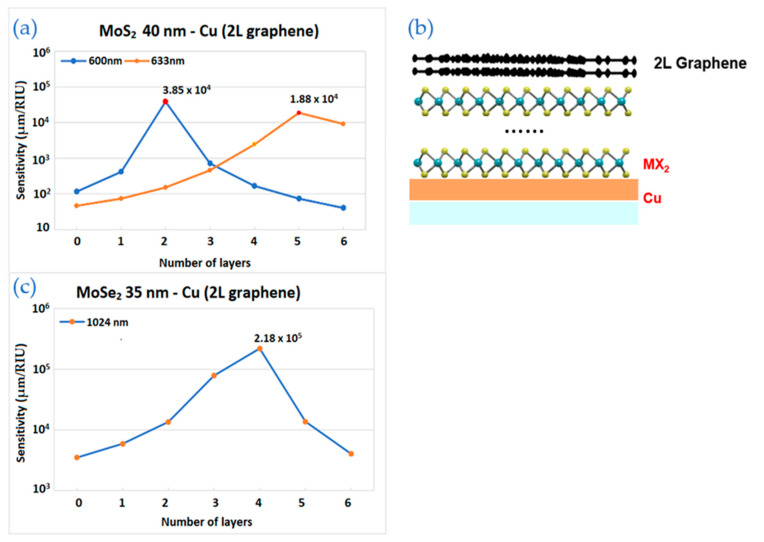
The sensitivity values of the enhanced model with 2 layers of graphene by TMDC layers and thickness of copper: (**a**) The wavelengths are 600 nm and 633 nm for MoS_2_ layers with 40 nm of copper; (**b**) the schematic structure; (**c**) The wavelength is 1024 nm for MoSe_2_ layers with 35 nm of copper.

The sensing performance with different TMDC layers were further investigated in addition to the configurations with graphene only. Configurations giving best improvement to the sensitivity were shown in the Figure 4, Figure 5 and Figure 6. First, the substrate only covered by four different TMDCs, without any graphene, was simulated. Because MX_2_ materials itself can protect copper surface from oxidation. In Figure 4, 3 configurations of 40 nm-thick copper covered by different 2D materials are shown.

Values for sensitivity has been calculated for all 4 TMDC materials for different thicknesses of copper and wavelengths. In Figure 4a, for the structure with MoS_2_, results for the excitation wavelengths 600 nm and 660 nm are shown. Number of layers are taken up to 5 layers. The 0 layer in the graph corresponds to structure without MoS_2_ layers on the surface and its value is indicated for comparison. The highest values of sensitivities for both incident wavelengths are around 9 × 10^4^ µm/RIU and correspond for 1 layer of MoS_2_. Another peak of sensitivity for 600 nm corresponds to 3 layers, and for 660 nm to 4 layers of MoS_2_. In Figure 4c, 40 nm Cu covered by different number of layers MoSe_2_ are shown. One can see from Figure 4 that for different excitation wavelengths, the highest SPR sensitivities are achieved with different optimum 2D material layers (600 nm with 3 layers of MoSe_2_, 600 nm with 7 layers and 785 nm with 5 layers). The highest sensitivity for this TMDC corresponds to 5 layers of MoSe_2_ and 785 nm as the incident wavelength.

In Figure 4d, thickness of copper film is 40 nm and coated by WSe_2_ up to 7 layers. There is only 1 excitation wavelength of 600 nm for sensitivity, because the results from other incident wavelengths are small compared to the result shown. For the excitation wavelength 600 nm and 5 layers of WSe_2_, the highest sensitivity for this structure was observed that equals to 3.98 × 10^4^ µm/RIU.

The best sensitivity value from the non-graphene results was 2.82 × 10^5^ µm/RIU shown in Figure 4c, which belongs to 5 layers of MoSe_2_ with 785 nm of excitation wavelength.

### 3.4. Sensitivity for TMDC and Graphene Enhanced Model

Previous configurations consisted of only thin film of copper covered by MX_2_ materials were studied. Combination of TMDC materials with graphene was taken to further enhance the sensitivity. Here we optimized the number of TDMC layers and incident wavelength. The number of graphene layers was fixed at 1.

Figure 5a illustrates the sensitivity calculations at 633 nm and 785 nm. For 1 layer of MoS_2_, the structure has an excellent result at 633 nm. Sensitivity value of 6.5 × 10^3^ µm/RIU is more than 100 times higher than without MoS_2_ layer. A gradual increase of sensitivity was observed with 785 nm incident wavelength, having its highest value for 7 layers.

In Figure 5c, the highest sensitivity quantity received is 3.85 × 10^4^ µm/RIU for 4 layers of MoSe_2_ on 40 nm Cu substrate at 785 nm. The sensitivities at 600 nm are also shown for comparison and its peak corresponding to 3 layers equals to 5.65 × 10^3^ µm/RIU.

Figure 5d shows the sensitivities of MoSe_2_ layers on 45 nm thick copper film with 633 nm excitation. The best sensitivity value corresponds to 1 layer of MoSe_2_. For other wavelengths, particularly for 660 nm, the curve has almost 4 times lower maximum sensitivity value.

Figure 6 shows the sensitivity results for MX_2_-graphene enhanced structure. The main difference from the structures above is 2 layers of graphene on top of thin layer of copper covered by TMDC layers. As shown in Figure 6, the optimal results do not always have the same wavelength and copper thickness.

Figure 6a shows the calculation by varying MoS_2_ layers with fixed 2 layers of graphene. The structure working at 600 nm and 633 nm has maximum sensitivities with 2 and 5 layers of TMDC, respectively. In Figure 6b, the schematic image of the structure is drawn.

In Figure 6c, there is a curve corresponding for 35 nm of Cu in near infrared wavelength range—1024 nm and with 2 layers of graphene. Sensitivity increases gradually starting from 0 up to 4 layers of MoSe_2_, where reaches its highest value—2.18 × 10^5^ µm/RIU and decreases with a further addition in the number of layers.

**Figure 7:**
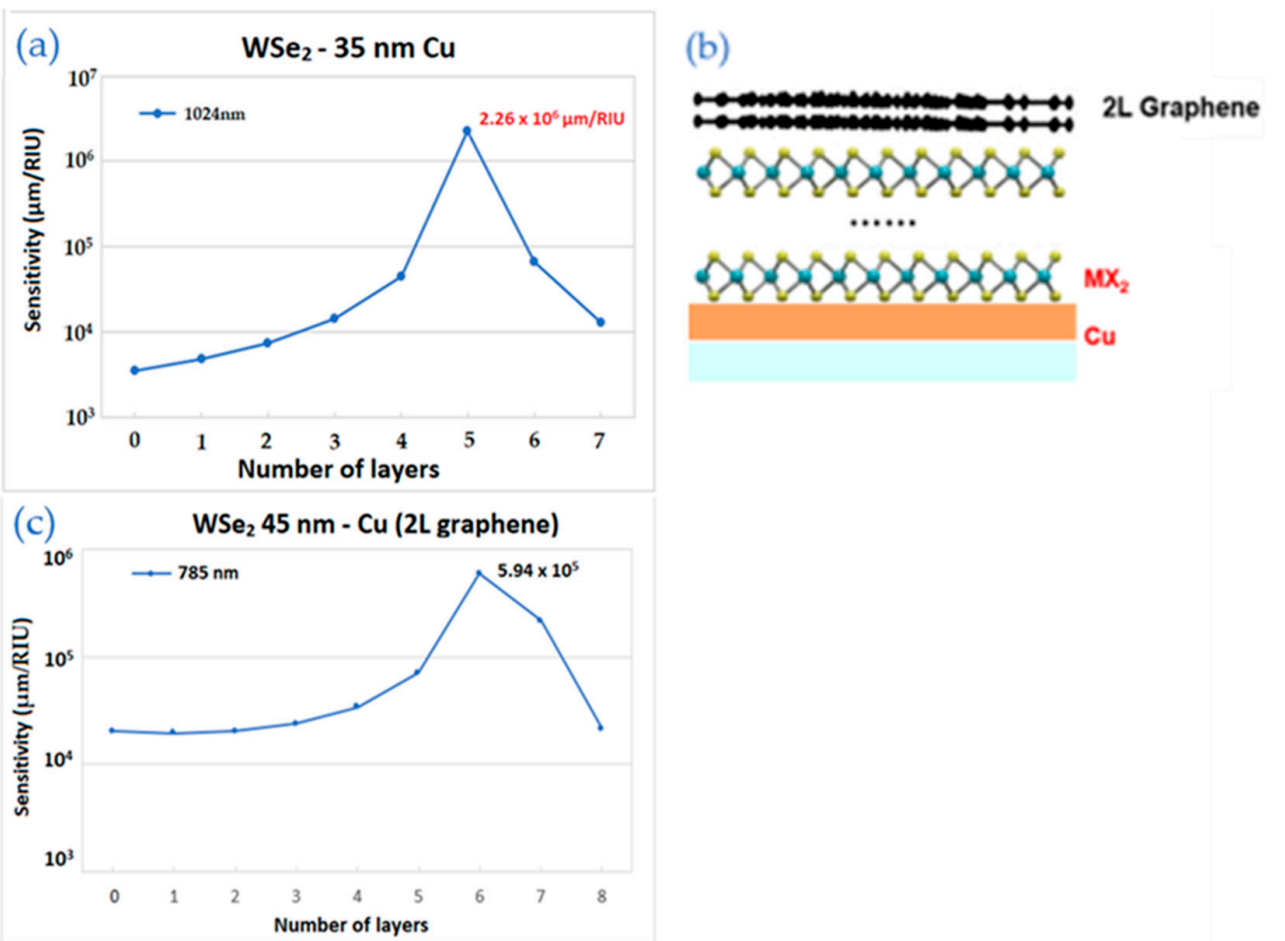
The sensitivity values of the enhanced model with 2 layers of graphene by TMDC layers and thickness copper: (**a**) The wavelength is 1024 nm for WSe_2_ layers with 35 nm of copper; (**b**) the schematic structure; (**c**) The wavelength is 1024 nm for WSe_2_ layers with 45 nm of copper.

Figure 7a shows the result for the configuration with 35 nm of copper film and 1024 nm near-infrared excitation wavelength. This SPR substrate exhibits the highest sensitivity observed so far, containing 5 layers of WSe_2_ and 2 layers of graphene. Its value equals to 2.26 × 10^6^ µm/RIU or 4.43 × 10^−10^ RIU/nm in another form.

Figure 7c shows the TMDC enhanced structure as in Figure 7a, but with 45 nm of Cu and 785 nm of excitation wavelength. Its maximum sensitivity corresponds to 6 layers of WSe_2_ and equals to 5.94 × 10^5^ µm/RIU, which is a high result compared to the structure without graphene.

## 4. Discussion

### 4.1. Comparison of Structures without and 1, 2 Layers of Graphene

By using different incident wavelengths from 600 nm up to near-infrared 1024 nm, variation of the complex refractive index was observed. Especially the ratio between the real and imaginary part has a big influence on optical properties of the material.

In Table 2, the maximum sensitivity values for structures of different number of graphene layers with 4 different TMDC materials with varying thickness of copper are shown. Here, sensitivity values for some structures are skipped, due to their low value even when compared to the structure without TMDC material.

The highest values of sensitivity are highlighted. First, comparing the results from the different thicknesses of thin copper film, it was found that only 35, 40 and 45 nm of Cu gives the best results. When the thickness of copper is too big, light cannot penetrate deep enough to excite surface plasmon polaritons on the other side of the metal. In addition, in the opposite case, when the thickness of copper is too small, there are not enough free electrons to create strong oscillations. Therefore, it was confirmed that the optimal thickness of thin copper film is from 35 up to 45 nm.

### 4.2. Best Results for Each Wavelength

These hybrid systems are highly dependent from excitation wavelength and incident angle of the beam. It is important to determine the best performance for every excitation wavelength used. Using different excitation wavelengths from visible and infrared regions causes a change in the complex refractive index. The ratio between real and imaginary part changes resulting in different optical properties of a plasmonic substrate.

Table 3 shows the best results for each wavelength. The maximum sensitivity values grow by applying bigger excitation wavelengths. It is caused by transmittance of graphene [26] and TMDC materials [27]. The highest sensitivities for 600 nm, 633 nm and 660 nm incident light corresponds to the structure with 1 layer of graphene or without any graphene. This indicates that for these relatively short wavelengths graphene transmits electromagnetic waves inefficiently. For wavelengths in the red or infrared region, the best values correspond to 5–6 layers of TMDC with 2 layers of graphene. These configurations still transmit light and show even better performances. The imaginary part in a refractive index related to absorption coefficient and can change the materials absorption properties with varying incident wavelength.

The best sensitivity observed corresponds to near infrared—1024 nm incident wavelength and its value equals to 2.26 × 10^6^ µm/RIU. This result is almost 4 times higher than for 785 nm excitation wavelength. The structure consists of 35 nm Cu, 5 layers of WSe_2_ and 2 layers of graphene. While 10 layers of graphene applied on Au surface, the performance of the plasmonic biosensor was improved by 25% [28].

In Table 3, only 3 TMDC materials from 4 used during simulations are included, which means that WS_2_ has poor plasmonic performance than other materials used. WS_2_ has relatively low maximum of sensitivity—5788 µm/RIU and it is almost 390 times lower than the best result observed.

## 5. Conclusions

We have demonstrated sensitivity calculations of the SPR system consisting of graphene with two dimensional TMDCs on Kretschmann configuration. The wavelength of incident light affects sensor’s performance. Five different wavelengths were chosen, both in visible and near-infrared region: 600 nm, 633 nm, 660 nm, 785 nm, and 1024 nm. TMM and Fresnel equations applied for phase change calculations.

To investigate the enhancement in sensitivity of such biosensors, we designed different models, such as graphene-MoS_2_, graphene-MoSe_2_, graphene-WS_2_ and graphene-WSe_2_. Moreover, we calculated systems only with graphene layers and then only with TMDC layers, to explore all the possible options. Even 2 layers of graphene, on top of thin copper film with a thickness from 35 up to 45 nm, can enhance the SPR resonance resulting better performance. TMDC materials can be used as a protection for copper surface from oxidation as graphene, so it can be used without graphene layers. We still observe some enhancement with only TMDC materials, and its highest value corresponds to 2.82 × 10^5^ µm/RIU. This value is from using 5 layers of MoSe_2_ with 40 nm of copper with 785 nm incident wavelength.

TMDC materials in combination with graphene gives even higher sensitivity results. The best value for sensitivity was 2.26 × 10^6^ µm/RIU, which is 1 order higher than the configuration without graphene.

With our system, we observed excellent sensitivity, and its detection limit equals to 8.8 × 10^−9^ RIU—lower than measurements with angle or wavelength interrogation [29]. This result corresponds to 5 layers of WSe_2_ and 2 layers of graphene, showing that combination of graphene with TMDC can enhance the sensitivity significantly.

These findings show that the thin copper film together with each of four TMDC materials could enhance the SPR effect and notably improve the sensitivity of the biosensors. Moreover, by varying the thickness of copper film and combining TMDC materials with graphene layers, it is possible to reach even higher values of detection sensitivity. These results are important for biosensors development, where sensitivity and stability are crucial for the effective use of final product.

## Figures and Tables

**Table 1:** Theoretical sensitivity (µm/RIU) calculation results for SPR biosensor with different thicknesses of copper without graphene and covered with 2 layers of graphene. The best values are marked in bold.

		Wavelength				
	Thickness of the Copper	600 nm	633 nm	660 nm	785 nm	1024 nm
Without graphene	30 nm	3.38	7.18	11.56	29.65	216.72
	35 nm	10.23	14.04	22.05	59.75	981.35
	40 nm	63.10	34.84	50.18	150.10	**4447.87**
	45 nm	277.06	174.17	193.18	**1164.60**	39.95
	50 nm	11.36	678.01	**6137.02**	372.84	30.87
2 layers of graphene	30 nm	3.34	6.82	11.17	30.01	270.72
	35 nm	11.57	15.04	23.93	67.40	**3498.87**
	40 nm	115.72	46.42	68.06	237.52	315.04
	45 nm	81.11	841.49	847.31	**20,139.79**	16.52
	50 nm	7.39	73.74	129.85	38.17	29.42

**Table 2:** Sensitivity values (µm/RIU) by graphene layers with MX_2_ materials. In the brackets number of MX_2_ layers and excitation wavelength are indicated.

	Thickness of Copper	Without Graphene (µm/RIU)	With 1 Layer of Graphene (µm/RIU)	With 2 Layers of Graphene (µm/RIU)
MoS_2_	45 nm	1.05 × 10^5^ (1 L, 600 nm)	6.52 × 10^4^ (6 L, 633 nm)	3.85 × 10^4^ (2 L, 600 nm)
MoSe_2_	35 nm	2.46 × 10^5^ (8 L, 1024 nm)		2.18 × 10^5^ (4 L, 1024 nm)
	40 nm	2.82 × 10^5^ (5 L, 785 nm)	3.84 × 10^4^ (4 L, 785 nm)	1.27 × 10^4^ (2 L, 600 nm)
	45 nm	2.89 × 10^4^ (1 L, 785 nm)	2.06 × 10^4^ (1 L, 633 nm)	8066 (1 L, 660 nm)
WS_2_	40 nm	4146 (10 L, 600 nm)	5788 (9 L, 600 nm)	
WSe_2_	35 nm	5155 (9 L, 1024 nm)	3.17 × 10^5^ (9 L, 1024 nm)	2.26 × 10^6^ (5 L, 1024 nm)
	40 nm	3.99 × 10^4^ (5 L, 600 nm)	1.03 × 10^4^ (4 L, 600 nm)	1.66 × 10^4^ (4 L, 600 nm)
	45 nm	346 (3 L, 633 nm)	1036 (3 L, 633 nm)	5.94 × 10^5^ (6 L, 785 nm)

**Table 3:** Highest sensitivity values (µm/RIU) for wavelengths: 600 nm, 633 nm, 660 nm, 785 nm, and 1024 nm.

Excitation Wavelength (nm)	Thickness of Copper (nm)	Type of TMDC	Number of TMDC Layers	Number of Graphene Layers	Sensitivity (µm/RIU)
600	40	WSe_2_	5	0	3.99 × 10^4^
633	45	MoSe_2_	1	1	2.06 × 10^4^
660	40	MoS_2_	1	0	9.09 × 10^4^
785	40	WSe_2_	6	2	5.94 × 10^5^
1024	35	WSe_2_	5	2	2.26 × 10^6^

## Data Availability

Data and MATLAB software underlying the results presented in this paper are not publicly available at this time but may be obtained from the authors upon reasonable request.

## Appendix A. Refractive Indices of Layers

Refractive index is one of the most important values for our calculations. The refractive index values for each layer were taken from reliable sources [30,31,32,33,34,35]. Refractive index values of Transition metal dichalcogenide (TMDC) were taken from the open database [30]. SF11 prism’s dispersion formula n_1_ as follows:

(A1) $$n_{1}=\left(\frac{1{.73759695\lambda }^{2}}{\lambda^{2}-0.013188707}+\frac{0{.313747346\lambda }^{2}}{\lambda^{2}-0.0623068142}+\frac{1{.89878101\lambda }^{2}}{\lambda^{2}-155.23629}+1\right)$$

where λ refers to wavelength in micrometers and in range of 0.37–2.50 microns.

The second layer is the BK7′s refractive index n_2_ can be found as follows [30]:

(A2) $$n_{2}=\left(\frac{1.03961212\lambda^{2}}{\lambda^{2}-0.00600069867}+\frac{0.231792344\lambda^{2}}{\lambda^{2}-0.0200179144}+\frac{1{.01046945\lambda }^{2}}{\lambda^{2}-103.560653}+1\right)$$

The complex refractive index of Chromium and copper thin film are from experimental data [31,32]. For graphene [33] the refractive index can be calculated as:

(A3) $$n_{6}=\left(3+i\frac{C_{1}}{3}\lambda \right)$$

where C_1_ = 5.446 µm^−1^. 1 layer of graphene has a thickness around 0.34 nm.

Fifth layer of the proposed SPR substrate is TMDC material and its complex refractive index and thickness are taken from experimental works as shown in Table A1 [34,35].

**Table A1:** The complex refractive indices of MoS_2_, MoSe_2_, WS_2_, and WSe_2_ with different excitation wavelengths from 600 nm to 1024 nm.

Type of TMDC	Thickness of Monolayer TMDC (nm)	λ = 600 nm	λ = 633 nm	λ = 660 nm	λ = 785 nm	λ = 1024 nm
MoS_2_	0.65	n = 4.3934	n = 5.0805	n = 4.9991	n = 4.6348	n = 4.5660
		κ = 1.2269	κ = 1.1723	κ = 1.2563	κ = 0.1163	κ = 0.1058
MoSe_2_	0.7	n = 4.7586	n = 4.6226	n = 4.4963	n = 4.2984	n = 3.8768
		κ = 1.1504	κ = 1.0063	κ = 0.9382	κ = 0.8225	κ = 0.3561
WS_2_	0.8	n = 3.5202	n = 4.8937	n = 4.4735	n = 4.0123	n = 5.0110
		κ = 0.6048	κ = 0.3124	κ = 0.2059	κ = 0.0399	κ = 0.2562
WSe_2_	0.7	n = 4.5039	n = 4.5501	n = 4.3357	n = 4.3655	n = 4.8690
		κ = 0.9340	κ = 0.4332	κ = 0.2532	κ = 0.0367	κ = 0.2444

The formula of complex dielectric constant is as follows:

ε = ε′ + ε″i (A4)

In the formula above, ε′ corresponds to energy inside of the material. And ε″ corresponds to the energy dissipation. It is possible to retrieve the refractive index with following relation:

ñ = √ε = n + κi (A5)

where the real part n corresponds to the phase velocity and the imaginary part κ known as the extinction coefficient and refers to the mass attenuation coefficient [36].
